# Distinguishing Physiological Ureter Uptake From an Involved Lymph Node in Staging Prostate-Specific Membrane Antigen (PSMA) Scans: Implications for Radiation Planning

**DOI:** 10.7759/cureus.63105

**Published:** 2024-06-25

**Authors:** Lauren Ching, Matthew Bourne, Tim Kearney, Karbi Choudhury, Alan L Zwart, Malika T Danner, Simeng Suy, Giuseppe Esposito, Sean Collins

**Affiliations:** 1 Radiation Oncology, MedStar Georgetown University Hospital, Washington, DC, USA; 2 Radiology, MedStar Georgetown University Hospital, Washington, DC, USA; 3 Nuclear Medicine, MedStar Georgetown University Hospital, Washington, DC, USA

**Keywords:** lymph node, ureter, positron emission tomography, ct urogram, ulcerative colitis, psma, prostate cancer

## Abstract

Prostate-specific membrane antigen (PSMA) imaging has become a mainstay diagnostic tool in staging unfavorable primary prostate cancer (PC) and identifying sites of recurrence in previously treated PC. One of the biggest pitfalls of PSMA imaging is rapid radionucleotide excretion in the urine via the​ kidneys, ureters, and bladder.​ The positron-emission tomography (PET) images obtained show increased radiotracer activity in these structures, which can occlude or even mimic true malignant disease. We describe the diagnostic challenges encountered in differentiating benign versus malignant disease with PSMA scans. A 78-year-old male presented ​to our outpatient radiation oncology office ​with high-risk prostate cancer. His medical history was significant for ulcerative colitis (UC). Magnetic resonance imaging (MRI) revealed an enlarged prostate and a Prostate Imaging Reporting and Data System (PI-RADS) class 4 lesion. A subsequent transperineal biopsy confirmed unilateral Gleason 8 adenocarcinoma.

A PSMA PET scan was read as increased uptake in the right prostate and a left external iliac node. The patient, having been initially informed of a positive lymph node metastasis, sought a second opinion,​resulting in​​ ​a CT urogram that revealed physiologic ureteral uptake. We were thus able to avoid lymph node radiation and morbidity to the surrounding bowel, already chronically inflamed with ulcerative colitis. This study ​demonstrates the ​potential for misinterpretation of PSMA uptake in the ureter as lymph node metastases. We discuss how peri-uretic activity can hinder accurate visualization of pelvic lymph node metastases. This study highlights the need for careful image interpretation of PSMA uptake patterns in order to avoid diagnostic errors and unnecessary radiation to ​at-risk​​ ​organs in prostate cancer management.

## Introduction

Prostate cancer is the third most prevalent cancer type in the United States, with an estimated 288,300 new cases among American males ​in ​2023 [1]. Imaging techniques play an integral part in ​the diagnosis and staging of prostate cancer, impacting treatment decisions for patients. For many years, the gold standard of diagnostic imaging modality for prostate cancer was the prostate MRI. The Prostate Imaging Reporting and Data System (PI-RADS) system is a radiological multi-parametric way to stratify how malignant a prostatic lesion appears on MRI​ ​by numbering them from 1 to 5. ​A ​PI-RADS 1 score indicates very low suspicion that the lesion is malignant, while​ a​ PI-RADS 5 score indicates a very high suspicion [2].

Recent advancements in imaging have been facilitated by the development of novel radiotracers. Prostate-specific membrane antigen (PSMA) is a transmembrane protein, the expression of which is significantly increased in prostate cancer, and ​as such ​it can be targeted with radiotracers for use in positron-emission tomography (PET) scans [3]. These radiopharmaceuticals have been effective in detecting primary and metastatic prostate cancer. ​The use of ​PSMA PET/CT improves the staging of prostate cancer, as it demonstrates superior sensitivity and specificity for detecting metastases compared to conventional imaging [4]. Furthermore, PSMA-targeted theragnostic agents​ ​have shown ​impressive ​potential in detecting and treating metastatic prostate cancer [5,6]. Agents like Gallium-68 (68Ga)-PSMA-11 and 18F-DCFPyL have been approved by the FDA for staging unfavorable prostate cancer patients [7] and evaluating sites of recurrence following a rising prostate-specific antigen (PSA) after curative treatment [8]. F18-labeled agents provide a higher sensitivity with a higher spatial resolution due to their greater positron yield and lower positron energy [9]. These attributes make PSMA scans a highly effective tool in diagnosing PSMA avid loco-regional and metastatic disease. In addition, PSMA scans may aid in patient-specific design of pelvic radiation treatment volumes [10].

However, despite the diagnostic advances for prostate cancer, PSMA-targeted PET imaging ​is not without its challenges. Most radiolabeled pharmaceuticals used for PSMA imaging are excreted by the renal system. This can result in increased radiotracer uptake in the kidneys, ureters, bladder, and urethra, which can obscure true malignant lesions ​adjacent to these structures. This ​detail ​becomes especially important when considering that most recurrences after prostatectomy occur in the prostatic fossa at the vesicourethral anastomosis, increasing the risk of missed detection​ [11]. Conversely, physiological​ uptake can​ also​ result in a false positive, where the reader mistakes bladder or ureteral activity for cancer. This study underscores the importance of detailed clinical understanding and careful interpretation of the imaging characteristics of PSMA scans in order to avoid potential misdiagnoses and unnecessary interventions in prostate cancer management [12].

## Case presentation

**Figure 1:**
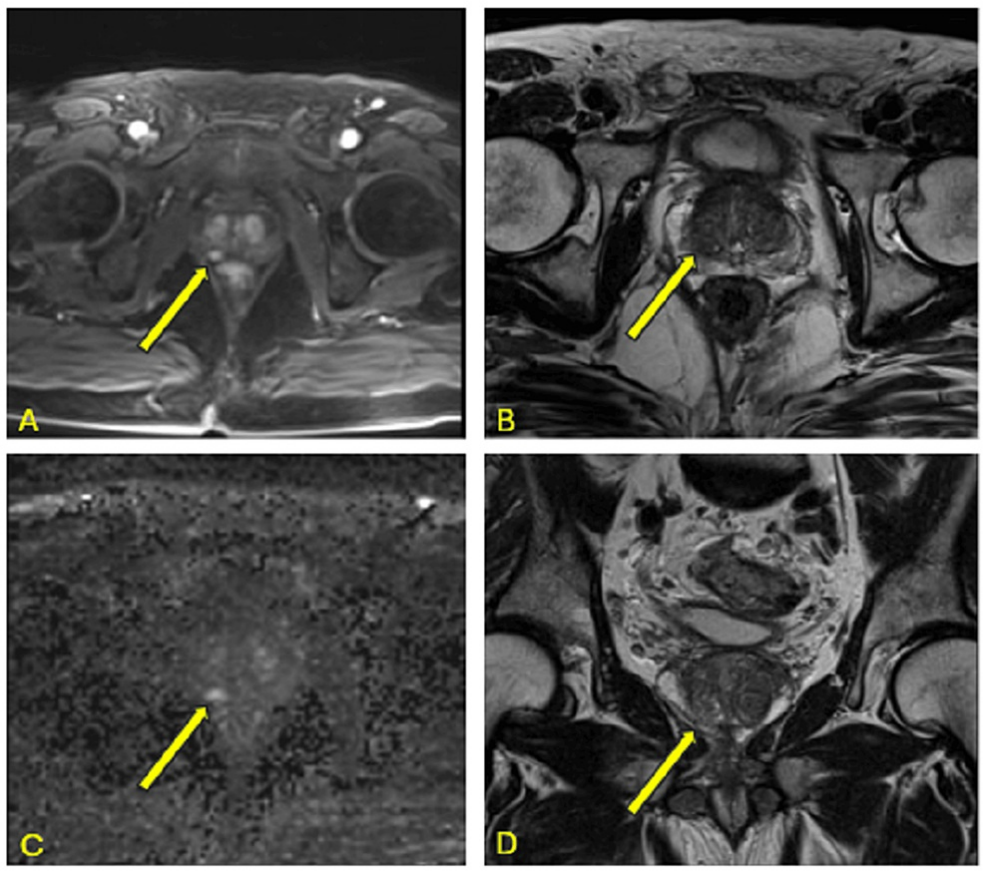
Multiple sequences of MRI prostate. Images show (A) axial T1-weighted MRI with volumetric interpolated breath-hold, (B) axial T2-weighted MRI, (C) axial MRI with diffusion-weighted imaging, and (D) coronal T2-weighted MRI.

A 78-year-old Caucasian male with a medical history of ulcerative colitis (UC) presented with stage IIC prostate adenocarcinoma (T1cN0Mx). His prostate-specific antigen (PSA) level upon screening was 4.2 ng/mL. Digital rectal examination (DRE) was unremarkable. Magnetic resonance imaging (MRI) showed an enlarged prostate measuring 50 cc with a right mid-peripheral zone (PZ) lesion (0.6 cm), with dynamic post-contrast enhancement and abutment of the capsule without extraprostatic extension, identified as PI-RADS 4 (Figures 1A-1D).

A transperineal (TP) biopsy was performed, where microscopic analysis showed unilateral Gleason 4+4=8 adenocarcinoma involving the right prostate. Notably, three out of 11 sampled regions were involved, with up to 50% core involvement.

**Figure 2:**
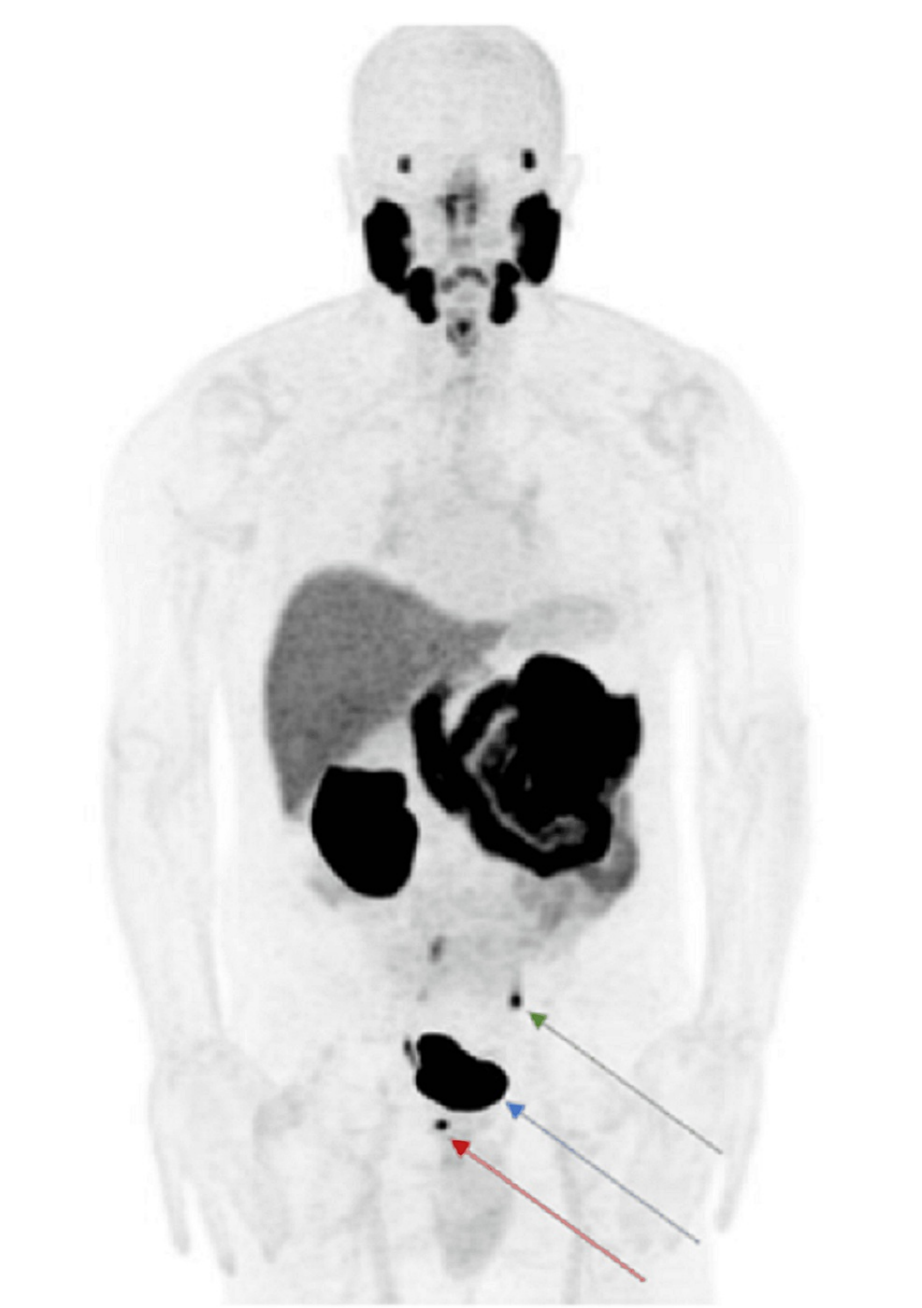
Frontal PSMA nuclear scan demonstrating intense radiotracer uptake in the prostate lesion (red arrow), bladder (blue arrow), and left ureter (green arrow). PSMA: prostate-specific membrane antigen

**Figure 3:**
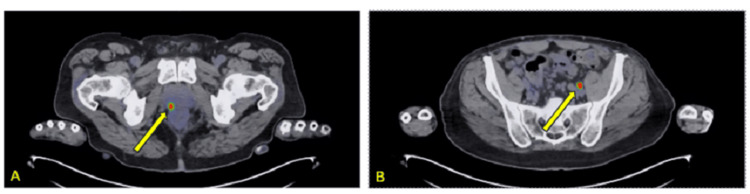
Axial PSMA scan used to​ assist​ with staging. Images (A) ​demonstrated ​increased uptake in the prostate and (B) ​demonstrated​ increased uptake in presumed left pelvic lymph node. PSMA: prostate-specific membrane antigen

Upon the patient’s initial presentation with high-risk prostate cancer, a staging prostate-specific membrane antigen (PSMA) piflufolastat F 18 (Pylarify; Billerica, MA: Lantheus) scan was obtained [7,8]. The patient had piflufolastat F 18 injected intravenously. Approximately 60 minutes later, PET imaging was performed from the top of the skull to the mid-thighs. Additionally, a limited non-contrast CT was performed for​ the​ purposes of attenuation correction and anatomic correlation. Of note, the patient did not eat for 6 hours prior to the scan, though he did remain hydrated. He was not given a diuretic prior to or during the scan. The scan was read by an in-house faculty nuclear medicine physician. The impression was that there was radiotracer accumulation in the right posterior medial peripheral zone, consistent with the known cancer (Figures 2, 3A). There was also increased radiotracer accumulation at a single point adjacent to the left iliac vasculature, thought to represent a prominent, though not enlarged, left internal iliac lymph node (Figure 3B).

**Figure 4:**
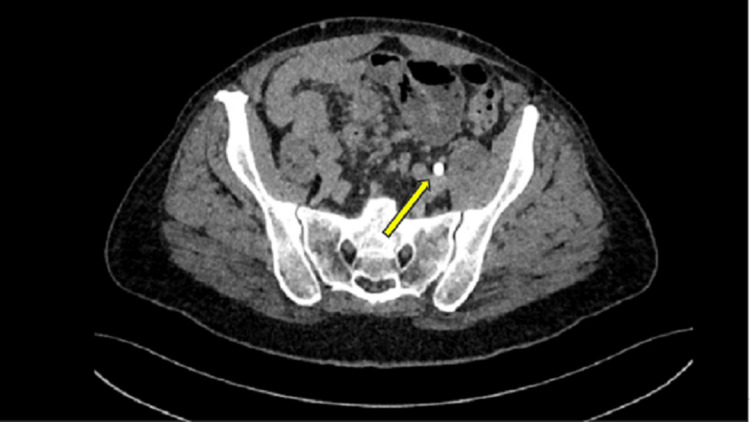
Axial CT with contrast, delayed depiction, with an arrow ​indicating ​the ureter.

**Figure 5:**
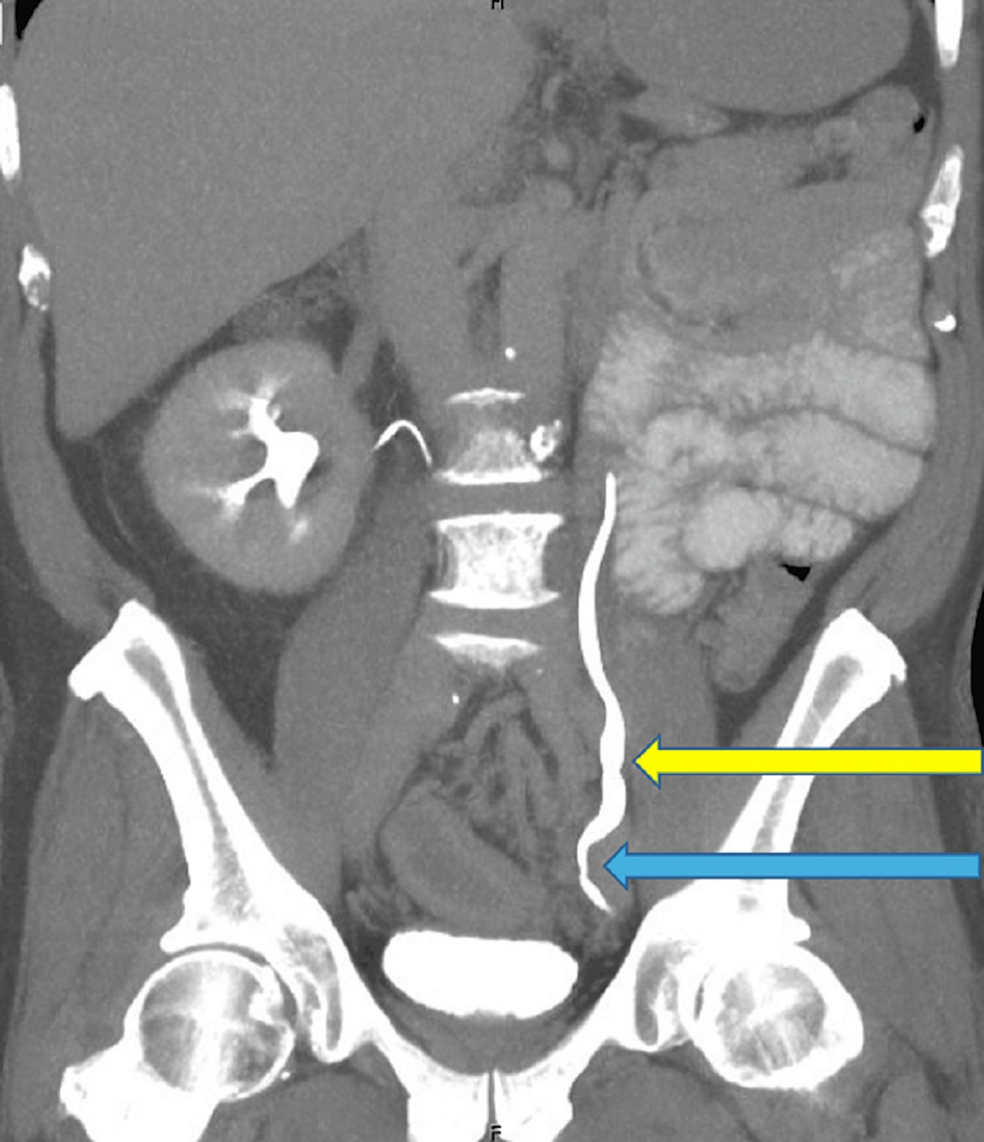
Coronal CT imaging with delayed contrast, displaying distal ureteral narrowing (blue arrow) resulting in mild hydroureter and tortuosity upstream (yellow arrow).

The radiation oncologist (SPC) recommended the patient receive 18 months of androgen deprivation therapy (ADT) combined with stereotactic body radiotherapy (SBRT) with supplemental pelvic radiotherapy, per institutional protocol [13]. The patient was advised that he would be at significant risk of high-grade gastrointestinal toxicity secondary to his ulcerative colitis and ​consequently ​sought a second opinion. The physician providing the second opinion ordered a CT Urogram. After injection of iodinated contrast, a dual-phase CT of the abdomen and pelvis was performed, and coronal and sagittal reformations were obtained. The scan revealed no evidence of pelvic lymphadenopathy to correspond with the findings seen on the previous PSMA scan (Figure 4). It showed narrowing of the left distal ureter (likely due to normal physiologic peristalsis), resulting in mild, temporary upstream hydroureter, radiographically corresponding with the increased uptake on the PSMA PET scan. Thus, the CT urogram revealed that what was initially thought to be a positive lymph node for metastasis turned out to be physiologic uptake in the ureter adjacent to the iliac vasculature (Figure 5).

T​his​​ ​patient’s case was reviewed at our institutional genitourinary (GU) tumor board, and the decision was made to proceed with long-course hormonal therapy with prostate SBRT alone to minimize late gastrointestinal side effects. The patient ​was amenable ​​to this. He underwent placement of fiducials into his prostate for interfractional prostate motion management as well as SpaceOAR Vue Hydrogel between his prostate and his rectum to reduce rectal dose [14,15]. His prostate and organs at risk (OARs) were contoured. The planning treatment volume (PTV) was planned to the 83% isodose line and treated to a total dose of 36.25 Gy over five fractions of 7.25 Gy [16]. Of note, he was concurrently treated with relugolix before, during, and after his radiation treatment [17]. He was seen by the faculty radiation oncologist for his end-of-treatment visit, where he complained of mild urinary symptoms and was advised to stay on his pretreatment tamsulosin and ​to ​continue​ a​ low-fiber diet. He was scheduled for routine follow-ups​ moving forward. The patient completed prostate SBRT and to date has not had high-grade GI toxicity. Overall, he was pleased with his care.

## Discussion

PSMA scans have greatly enhanced our ability to localize newly diagnosed and recurrent prostate cancer and​ to​ assess the extent of ​the malignancy​ [3,4]. Especially in the early stages of disease, accurate assessments of disease burden can be crucial for determining the appropriate approach to curative therapy. Our case exemplifies one of the common pitfalls of radionucleotide imaging - the false positive read caused by physiologic urinary excretion. Our patient was found to have radiotracer accumulation (standardized uptake value {SUV}: 13.19) in a presumed non-enlarged left-sided pelvic lymph node, which upon further diagnostic investigation, was likely to be urinary stasis caused by normal ureteral peristalsis. Treatment planning based on this scan alone would have resulted in whole-pelvis radiation and hormonal therapy-increasing the bowel toxicity ​in a male ​already suffering ​​​from ​​ulcerative colitis. Unfortunately, urine radiotracer uptake can yield a similar uptake value as prostate cancer. A recent retrospective study that assessed 57 cases of biochemical recurrence after curative treatment found that the average SUV of recurrent prostate lesions found on 68Ga-PSMA-11 was 14±7.5 [18]. Potential approaches to reducing the impact of urine activity include​ prophylactic​ diuretic administration, large delays in scanning and/or multiple scan times, or as ​in​ this study, additional imaging (CT urography and/or MRI). ​However, these approaches may not adequately address the problem ​as well as ​add unnecessary costs and delays in management [19].

​​​Inflammatory bowel disease (IBD), such as​ Crohn's disease or​ ulcerative colitis, is a chronic idiopathic inflammatory disorder that has historically been considered a soft contraindication to radiation therapy [20,21]. The chronic inflammation of the bowels can result in fistula formation, strictures, poor nutrient absorption, and diarrhea. As such, efforts are traditionally taken to limit the exposure of these patients to pelvic radiation therapy, which would further increase their risk of complications. It is thought that IBD amplifies the toxicity of radiation in a synergistic effect; however, ​the mechanism by which this occurs remains unclear.

If it is​ absolutely​ necessary to administer pelvic radiation to someone with IBD,​ the​ ​existing ​literature suggests that doing so while the IBD is dormant may offer the best chance of sparing extra toxicity. Data has emerged assessing the outcomes of these patients after definitive stereotactic body radiotherapy (SBRT) for prostate cancer. A recent single-institutional report of 31 patients with IBD who were treated with SBRT for prostate cancer had excellent quality of life outcomes at one year [22]. Moreover, a control-matched review of 39 males with inactive IBD who were treated with prostate SBRT showed that IBD was not associated with increased odds of late​ grade ≥​2 GI or GU toxicity (median follow-up time ​of ​89 months). There was, however, an increased acute risk of low-grade GI/GU toxicity in those with dormant IBD [23]. This data offers encouraging insight into the outcomes of those who received radiation with dormant IBD; however, it is still generally recommended to avoid pelvic radiation, if possible, during an acute IBD flare-up.

The ureter is a retroperitoneal structure that is susceptible to radiation toxicity. Radiation-induced changes to the ureters include endarteritis, ischemia, tissue contraction, and fibrosis. This can lead to significant genitourinary impairment in the form of ureteral atrophy and stenosis. These conditions impair tissue healing and lead to ureter​al​ atrophy and contraction, ultimately resulting in ureteral stenosis [24-26]. Additionally, case reports have shown evidence that inflammation of the retroperitoneal bowel structures (e.g., ascending or descending colon) can increase risk of ureteral strictures [25]. Though there is a paucity of literature assessing the odds of developing ureteral strictures with IBD, it can be extrapolated that inflammation of the retroperitoneal colon, adjacent to the ureter, can increase the risk of ureteral strictures. There is limited data ​assessing ​the rates of ureteral injury with definitive prostate SBRT; however, there is evidence to suggest that traditional or moderately hypofractionated external beam radiotherapy (EBRT) can convey an increased risk of ureteral injury by 0.25% per year for up to 25 years [26]. It is therefore prudent to avoid pelvic radiotherapy if possible, especially when the patient’s comorbidities can increase the risk of radiation-associated toxicity to pelvic structures​ such as ​the bowel and ureters.

Definitive treatment of prostate cancer that has spread to the pelvic lymph nodes requires higher doses of radiation to the pelvis, sometimes incorporating a simultaneous-integrated boost (SIB) to the PET-positive lymph node [27]. A false-positive lymph node on a PSMA, as laid out in the present case, can therefore expose the patient to unnecessary treatment risk and morbidity. Instead of radiation to the prostate​ alone, these patients usually receive radiation to the prostate and the pelvic lymph nodes [28]. Specifically, they can receive anywhere between 45 Gy and 54 Gy to the pelvic lymph nodes as well as 19.5-21 Gy to the prostate with SBRT [14,29]. A positive pelvic lymph node can result in hormonal therapy for 24 months or longer, as opposed to the more tolerable 18 months if the cancer is confined to the prostate [30]. In addition, intensification with secondary hormonal agents would be considered [30]. Because we were able to discern that our patient’s PSMA scan yielded a false-positive lymph node, we were ​only able to ​​avoid potentially morbid pelvic treatment ​and ​limit systemic therapy to ​a shorter, less intense course of hormonal therapy [30]. Due to adverse side effects of ​androgen deprivation therapy, it is advisable to avoid ​extended courses of hormonal ​therapy in elderly males.

There are currently three PSMA-targeting radiopharmaceuticals approved by the FDA, the most recent of which is 18F-rhPSMA-7.3 (Posluma; Oxford, UK: Blue Earth Diagnostics Ltd). A 2021 phase 1, open-label study assessed the biodistribution of 8F-rhPSMA-7.3 in six healthy volunteers at different time points after injection. Blood and urine volumes of the radiotracer were taken in regular increments between 30 seconds and 255 minutes after injection. The first post-injection urine sample taken 90 minutes after injection showed an average urinary excretion of 7.2% (range: 4.4-9.0%) [31]; this is significantly lower than ​the ​values noted for 18F-DCFPyL (11%) and 68Ga-PSMA-11 (11%) in the first 2 hours [32]. Because most PET protocols suggest imaging approximately 1 hour after administration of the radiotracer, we can infer that PET/CT performed with 18F-rhPSMA-7.3 has less diagnostic interference with urinary structures due to slower urinary excretion of 18F-rhPSMA-7.3 compared to other radiopharmaceuticals.

The efficacy of 18F-rhPSMA-7.3 PET/CT scans in detecting prostate cancer has been assessed in two phase III clinical trials. The LIGHTHOUSE trial looked at males with newly diagnosed unfavorable prostate cancer who were planned to undergo a prostatectomy with pelvic lymph node dissection (PLND) [33,34], and the SPOTLIGHT trial assessed the detection rate of biochemically recurrent prostate cancer [35-37].​ As in the present case, patients in these trials were not given diuretics, and PET imaging was performed 50-70 minutes after administration of the radiopharmaceutical. A post-hoc analysis of these two trials examined 712 of the PSMA PET scans performed on their​ patients. By expert opinion, 96% of the scans were considered to be unobscured by physiologic urinary activity, and ureteric activity was absent in >50% of scans altogether [38,39]. While ​these results​ ​are promising, ​it is important to bear in mind that these​ studies​ ​were​ not designed to prove 18F-rhPSMA-7.3 superiority over other radiopharmaceuticals, ​and ​so SUV comparisons between modalities must be made with caution.

## Conclusions

This study reinforces the challenges of ​singularly ​using PSMA PET scans in prostate cancer management. It draws attention to the diagnostic ​difficulties ​clinicians may face when differentiating between periuret​er​al activity ​and malignancy, which ​can hinder accurate visualization of pelvic lymph node metastases. Erroneous interpretations can impact healthcare outcomes regarding radiation therapy toxicities. A thorough understanding of the various patterns of PSMA uptake is vital ​for minimizing ​errors in image interpretation. An approach ​that concurrently utilizes​​ ​CT urograms​ can offer a valuable adjunct to assist in cases of a difficult diagnosis. The case presented here underlines the necessity of careful interpretation and the importance of developing multimodal approaches to ensure proper treatment for patients with prostate cancer.
